# Robust Assembly of Cross-Linked Protein Nanofibrils into Hierarchically Structured Microfibers

**DOI:** 10.1021/acsnano.2c03790

**Published:** 2022-07-29

**Authors:** Xinchen Ye, Antonio J. Capezza, Saeed Davoodi, Xin-Feng Wei, Richard L. Andersson, Andrei Chumakov, Stephan V. Roth, Maud Langton, Fredrik Lundell, Mikael S. Hedenqvist, Christofer Lendel

**Affiliations:** † Department of Fibre and Polymer Technology, KTH Royal Institute of Technology, Teknikringen 56-58, SE-100 44, Stockholm, Sweden; ‡ Department of Engineering Mechanics, KTH Royal Institute of Technology, Teknikringen 8, SE-100 44, Stockholm, Sweden; § Deutsches Elektronen-Synchrotron DESY, Notkestraße 85, D-22607 Hamburg, Germany; ∥ Department of Molecular Sciences, SLU, Swedish University of Agricultural Sciences, BioCentrum, Almas allé 5, SE-756 61, Uppsala, Sweden; ⊥ Department of Chemistry, KTH Royal Institute of Technology, Teknikringen 30, SE-100 44, Stockholm, Sweden

**Keywords:** protein nanofibrils, amyloid, hierarchal assembly, cross-linking, flow-focusing

## Abstract

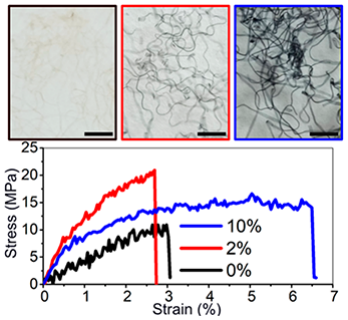

Natural, high-performance fibers generally have hierarchically organized nanosized building blocks. Inspired by this, whey protein nanofibrils (PNFs) are assembled into microfibers, using flow-focusing. By adding genipin as a nontoxic cross-linker to the PNF suspension before spinning, significantly improved mechanical properties of the final fiber are obtained. For curved PNFs, with a low content of cross-linker (2%) the fiber is almost 3 times stronger and 4 times stiffer than the fiber without a cross-linker. At higher content of genipin (10%), the elongation at break increases by a factor of 2 and the energy at break increases by a factor of 5. The cross-linking also enables the spinning of microfibers from long straight PNFs, which has not been achieved before. These microfibers have higher stiffness and strength but lower ductility and toughness than those made from curved PNFs. The fibers spun from the two classes of nanofibrils show clear morphological differences. The study demonstrates the production of protein-based microfibers with mechanical properties similar to natural protein-based fibers and provides insights about the role of the nanostructure in the assembly process.

Many biological materials found in nature are generated through the self-assembly of building blocks with a well-defined organization at multiple length scales, rendering their extraordinary mechanical properties and advanced functionalities. Silk fiber^1^ is an example of such materials having defined structures, where nanosized building blocks are structured first into micro- and then macrofibers with a high order of orientation along the fiber axis. However, such hierarchical assembly of molecular components into high-performance micrometer-scaled bulk materials has been challenging to mimic in artificial systems without losing the extraordinary mechanical properties of the nanoscale building blocks.^2,3^ Improved knowledge is needed in order to control the assembly process and allow the design of bio-based material for a wide range of applications.

With this goal in mind, the assembly of protein molecules into highly ordered, amyloid-like protein nanofibrils (PNFs) offers very interesting opportunities. PNFs form spontaneously *in vitro* under proper conditions from various protein sources, *e*.*g*., the bovine whey protein β-lactoglobulin in pure form,^4,5^ or in whey protein isolate,^6,7^ potato protein,^8^ hen-egg lysozyme,^9^ and various legume protein sources.^10,11^ This makes the PNFs interesting as building blocks for large-scale production of sustainable materials.^12^ Amyloid-like fibrils have already successfully been demonstrated as materials for, for example, drug delivery, solar energy conversion, and biosensors.^13,14^ A demonstrated advantage of the synthesis of PNFs is that the morphology, *i*.*e*., their nanoscale structures, sizes, and curvatures, can be controlled by altering the starting material and the fibrillation conditions.^6,8,10,11^ Thus, using PNFs as building blocks is an attractive road for tailoring material properties. Different spinning methods have been explored to further assemble the PNFs at the microscale level, such as wet-spinning^15^ and microfluidic techniques.^16,17^ These methods have also proved successful in assembling silk fibrils^18^ and cellulose nanofibrils.^19−22^ Among the methods, the microfluidic technique with hydrodynamic forces applied to nanofibrils for alignment has been demonstrated as a promising method for controlled microfiber formation. The hydrodynamic forces are created by an extension flow, where the flow velocity is higher than the core flow of nanofibrils to achieve shear-induced alignment. It has been reported that, with a higher speed of the focusing flow, the fiber obtained a higher fibrillar alignment and a higher Young’s modulus.^16^

In our previous study, PNFs from whey protein isolate (WPI) having two distinct nanoscale morphologies, straight and curved, were assembled into hydrogel microfibers using the flow-focusing method.^17^ The mechanical properties of the fibers assembled from pure PNFs were found to be strongly dependent on the nanofibril morphology. Microfibers having a modulus up to 288 MPa and a strain at break of *ca*. 1.5% could be produced from curved PNFs, while fibers from straight PNFs were not strong enough to be collected from the bath at the end of the spinning process (see Figure S1 for the description of the setup). We hypothesized that the curved PNFs resulted in a higher degree of entanglement and thereby a higher density of physical cross-links.

In this study, a nontoxic chemical cross-linking agent, genipin, was used to investigate the impact of cross-linking on the mechanical properties of the spun microfibers from curved whey PNFs and also enabling the production of microfibers from straight PNFs. Genipin, extracted from the gardenia fruit, is a biodegradable reagent that has been evaluated as a substitute for toxic aldehyde cross-linkers (*e*.*g*., formaldehyde and glutaraldehyde).^23−25^

## Results and Discussion

### Cross-Linking PNFs with Genipin

**Figure 1:**
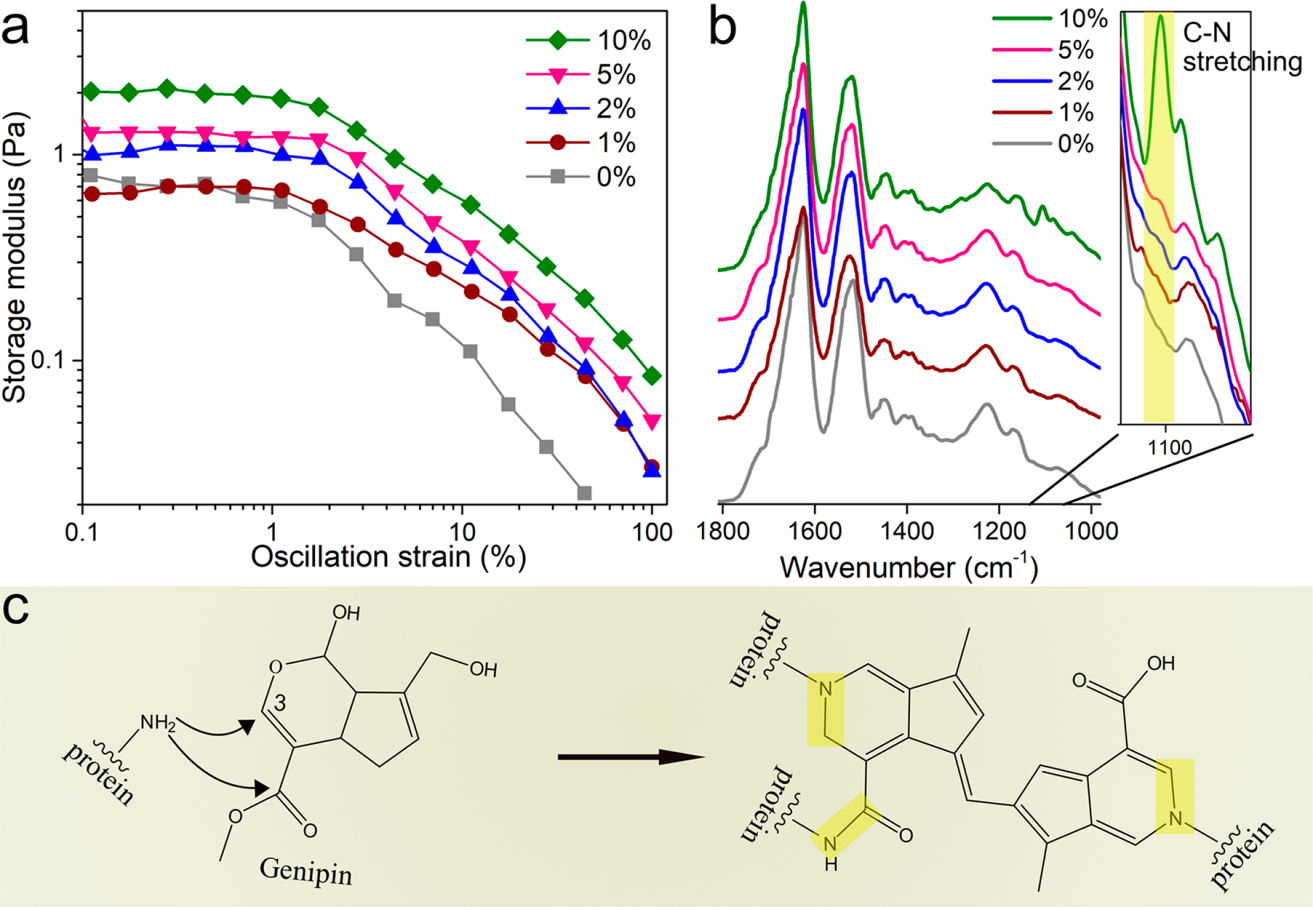
(a) Storage modulus of the cross-linked and non-cross-linked PNF networks (curved fibrils, 16 g/L) *versus* oscillatory strain. (b) IR spectra for these PNF–genipin samples. The amount of added genipin in relation to the increase in C–N stretching is indicated. (c) Protein cross-linking mechanism by genipin in acidic conditions. The generated new C–N bonds in the product were highlighted in yellow as examples.

The cross-linking effect of genipin on the curved PNF network was studied using rheology and IR spectroscopy. Various amounts of genipin powder were mixed into the PNF suspension and incubated at 50 °C for 14 h before the measurements, as the reaction is more efficient at elevated temperatures (50–60 °C).^26^ The storage modulus of all PNF suspensions was higher than the loss modulus (not shown), suggesting that the PNF suspensions were more elastic than viscous.^27^ The storage modulus–oscillation strain curves of these PNF suspensions, with and without genipin, showed a similar shape (Figure 1a), indicating a similar behavior when subjected to an oscillating strain of 0.1% to 100%. The storage modulus of all samples was relatively stable at strains lower than *ca*. 1% and decreased with increasing strain with a similar slope. The addition of 1 wt % genipin (with respect to the PNF content) displayed a small effect on the modulus–strain curve. An increase in the amount of genipin (>2 wt %) shifted the curves to higher storage modulus, and the modulus increased with increasing genipin content in the PNF suspension. This suggests the formation of a strong network with a strength dependent on the amount of genipin present in the system.

The formation of genipin cross-links between nanofibrils was further supported by the IR spectra of the PNF–genipin samples (Figure 1b). It has been reported that the primary amino groups attack the genipin C-3 carbon, forming heterocyclic amines, which further associate, generating cross-linked networks with short genipin oligomer bridges as illustrated in Figure 1c.^28−30^ The reaction is also accompanied by nucleophilic substitution of the ester group on genipin by primary amine groups in acidic conditions. Both reaction mechanisms generated new C–N bonds, observed in the IR results. The absorbance peak at *ca*. 1100 cm^–1^, stemming from the C–N stretching, is most prominent in the 10 wt % genipin–PNF sample and but also visible as a shoulder in spectra of the PNF samples cross-linked with 2 and 5 wt % of genipin.^29^ The presence of the peaks in all spectra with added genipin is confirmed by the second derivative spectra (Figure S2). The increase of the peak intensity correlated well with the rheology results described above, suggesting the formation of more cross-links. However, it is difficult to determine the amount of genipin reacted with protein to form oligomer bridges and the remaining unreacted amount. The amide I and II regions (1500–1700 cm^–1^) in the IR spectra of the PNFs with and without genipin did not show significant peak shifts or shape variations, indicating that the PNF structures remained intact in the cross-linked network. Genipin is expected to primarily react with amine groups (*i*.*e*., lysine side chains). The lysine content in the WPI PNFs is between 5% and 10%, depending on which parts of the β-lactoglobulin sequence are present in the final PNFs. This gives a genipin:lysine ratio between 0.5 and 1.5 for 10% genipin. Hence increased cross-linking is expected up to 10% genipin.

### Mechanical Properties of the Cross-Linked PNF Microfiber

**Figure 2:**
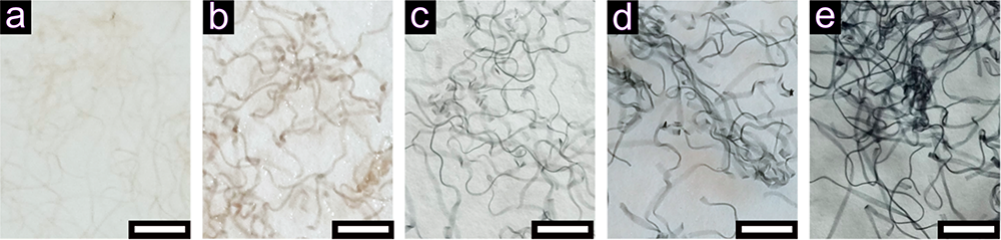
Images of the nanostructured protein microfiber assembled from 20 g/L curved PNFs in acetate buffer solution (pH 5.2) without genipin (a) and cross-linked with 1 (b), 2 (c), 5 (d), and 10 (e) wt % of genipin after 14 h of incubation at 50 °C. The scale bar is 300 μm.

The microfiber was formed using a double-focusing millimeter-scale device with a core flow (Q1) of the curved PNF suspension and two sheath flows (Q2, Q3) for focusing (Figure S1a). Genipin was added directly in the core flow mixed with a PNF suspension rather than in the second sheath or bath solution, to maximize the interactions between PNFs and genipin used in the system. The microfiber without the addition of genipin remained colorless after 14 h of incubation at 50 °C; see Figure 2a. In contrast, the microfibers with genipin obtained a brown/dark green color after the same treatment (Figure 2b–e), which has been previously reported and suggested to be a result of the reaction between the primary amines and genipin.^26,31^ The color of the fiber darkened with an increasing amount of genipin added in the PNF suspension, indicating that more genipin reacted with the PNFs. This observation correlated well with the rheology and IR results (Figure 1).

**Figure 3:**
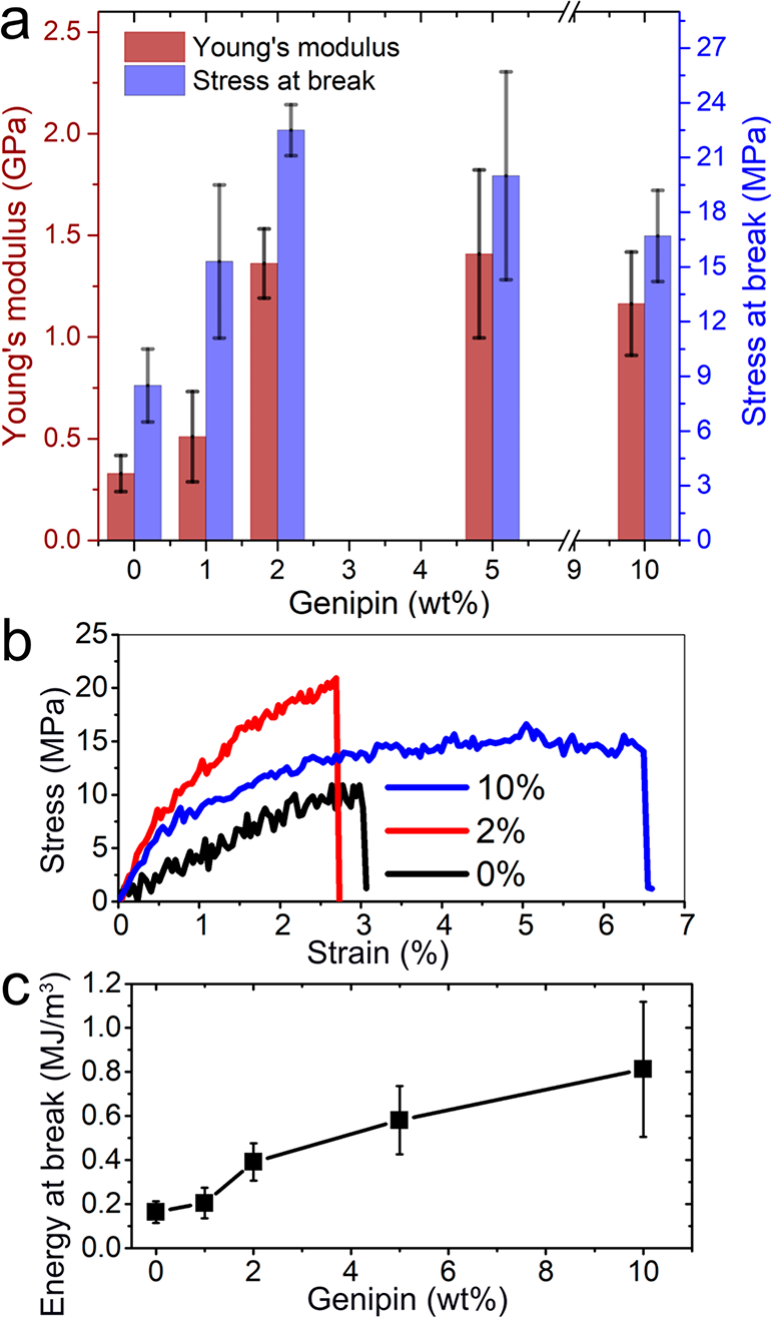
(a) Mechanical properties of the microfibers spun from curved PNFs (20 g/L) with genipin, measured at 50% relative humidity. (b) Representative stress–strain curves of microfibers spun from curved PNFs with 0, 5, and 10 wt % genipin. (c) Plot of energy at break relative to the genipin content in the fibers.

The formation of the cross-links within the PNF networks also improved the microfiber’s mechanical properties (see Figure 3). The presence of a low amount of genipin (<2 wt %) increased mainly the Young’s modulus (from now on referred to as modulus) and the stress at break of the fiber, while a higher amount of genipin addition (*i*.*e*., 5 and 10 wt %) increased the strain at break of the fiber (Figure 3a and b). The microfiber spun solely from a PNF suspension showed a modulus of *ca*. 0.33 GPa (which is in the expected range^17^) and stress at break of *ca*. 8.5 MPa. The higher stress at break and elongation at break (3%) obtained here compared with those reported in the previous work (3 MPa and 1.5%, respectively) could be the effect of the slightly higher PNF concentration used herein and the 4-day conditioning before the tensile tests (in previous work^17^ the fibers were tested in the dry state). The modulus and the stress at break increased to 0.51 GPa and 15 MPa, respectively, when 1 wt % of genipin was added into the suspension before the spinning process. A more significant increase of the modulus and stress at break occurred with the addition of 2 wt % genipin, resulting in 1.4 GPa and 22 MPa (Figure 3a). However, a higher concentration of genipin did not further increase the modulus and stress at break, which remained at 1.4 GPa and 20 MPa with 5 wt % genipin and slightly decreased to 1.2 GPa and 17 MPa, respectively, at 10 wt %. In contrast, the strain at break of the fiber cross-linked with 10 wt % genipin increased 2 times, reaching *ca*. 6%; see Figure 3b. The energy at break of the fiber with 10 wt % genipin (0.81 ± 0.03 MJ/m^3^) was 2 and 5 times that of the fiber with 2 wt % (0.39 ± 0.09 MJ/m^3^) and without genipin (0.16 ± 0.05 MJ/m^3^), respectively (Figure 3c).

The mechanical performance of the produced micrometer-scale materials is governed by the arrangement of the building blocks and the interfaces that join these building blocks.^2,3^ The addition of genipin increased the cohesion between the nanofibrils (Figure 1), formed stronger fibril interfaces that facilitated load transfer during testing, and resulted in a higher modulus and stress at break (Figure 3). The finding that higher amounts of genipin (>5 wt %) do not lead to a further increase in modulus and strength suggests a limit for the cohesive forces despite the fact that more genipin has reacted with the PNFs. This behavior differs from the rheology of bulk samples (Figure 1a). The packing of the PNFs that results from the flow-assisted assembly may not allow further reactions between the bound genipin groups to form interfibrillar cross-links. Moreover, the higher extensibility of the microfiber at high genipin content suggests increased plasticity of the material (Figure 3b and c). Since no plasticizer was added, this effect could result either from unreacted genipin in the fiber or from an enhanced water uptake in the cross-linked fiber. Water has a strong plasticizing effect on bio-based materials, which is evident, for example, from our previous work on cellulose fibers (see Figure 3b in Mittal *etal*.^19^). Previous work has demonstrated that the addition of genipin significantly increases the water uptake in various biopolymer materials.^32−34^ To investigate if the whey PNF materials show the same behavior, we measured the water uptake in samples with 5% or 10% genipin (reacted under similar conditions to those in the microfibers) and compared it with the material without genipin. The water content was quantified by thermogravimetric analysis (TGA) (Figure S3) and was found to be 6.2%, 5.6%, and 5.1% for the samples with 0%, 5%, and 10% genipin, respectively. Hence, the water uptake is lower in the samples reacted with genipin, which is expected for a system with a higher degree of cross-linking. The results show that increased water content cannot be the origin of the plastication effect at 5–10% genipin content and suggest that genipin itself is responsible for the observed behavior. Changes in mechanical properties similar to what we observe (*i*.*e*., decreased modulus and stress at break and increased strain at break) have been reported for films made from starch and potato protein with genipin added. and one of the explanations for this behavior suggested in that work was that genipin could act as a plasticizer.^35^ Another study on elastin cross-linked with genipin also reported that the compressive modulus levels out at 7–10% genipin.^36^ Further exploration of the mechanism behind this behavior requires detailed experimental analysis using methods that can quantitatively distinguish between genipin in different states. At least four states may exist in the fiber: (i) monomeric, (ii) oligomeric, (iii) reacted with PNFs, and (iv) reacted with PNFs and cross-linked. The two first states may act as plasticizers, while the effect of state (iii) on the mechanical properties is difficult to predict.

### Effect of PNF Morphology and Sheath Flow Rate on the Mechanical Properties of the Microfiber

The PNF suspension used to assemble microfibers described so far contained the PNFs that were short and curved with a mean fibril end-to-end length of 0.3 μm (Figure S4b). By changing the initial WPI concentration, straight and longer PNFs with a mean fibril end-to-end length of *ca*. 1.2 μm could be produced (Figure S4a).^6,17^ The persistence length of the straight fibrils was previously reported to be *ca*. 1960 nm, which was almost 50 times higher than that for the curved fibrils (41 nm).^17^ However, the hydrogel fibers assembled from only straight PNFs were not strong enough to overcome the surface tension at the liquid–air interface when pulled out from the acetate bath, potentially due to a lack of fibril–fibril entanglements.^17^ To strengthen the interactions between the straight PNFs, 10 wt % of genipin was added into the spinning suspension to cross-link the fibrils and improve the mechanical properties of the final fibers. However, the hydrodynamic focusing of the straight PNF suspension was not successful using the same flow parameters used previously for the curved fibrils. The core flow of the suspension did not remain as a stable flow parallel to the flow channel but tended to twist/oscillate and clog the channel. This could originate from the viscosity difference between the straight and curved PNF suspensions (Figure S5). Evidently, the suspension properties with the straight PNFs were not optimal for a stable flow and a resulting continuous ejected microfiber.^21,37^ The unstable flow and oscillations were addressed by increasing the flow rate of the second sheath flow (Q3) to 33.9 mL/h (from 24.9 mL/h). The spinning of the curved PNF suspension was repeated at the elevated Q3 sheath flow rate to compare the final fibers.

**Figure 4:**
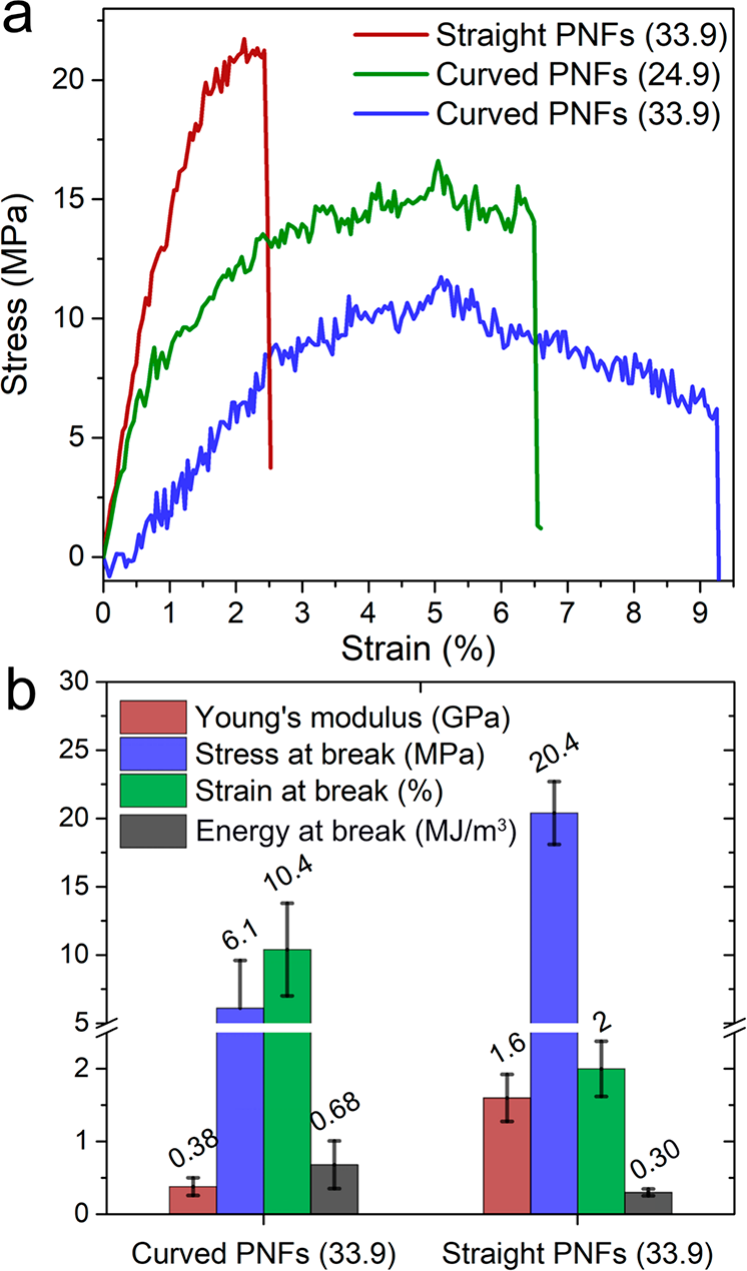
(a) Stress–strain curves of microfibers assembled from straight and curved PNFs with 10% genipin at different sheath rate (illustrated as the number in the legend, unit: mL/h). (b) Mechanical properties of the fibers at a sheath rate of 33.9 mL/h.

The stress–strain curves of the fiber assembled from straight PNFs showed a stiffer behavior compared with that of the fibers spun from curved PNFs (Figure 4a). The microfiber from straight PNFs had a modulus as high as 1.6 GPa and stress at break of *ca*. 20 MPa, which is around 4 times higher than that for the microfibers from the curved PNFs (0.38 GPa and 6 MPa, respectively); see Figure 4b. However, fibers from curved PNFs showed a higher strain at break of *ca*. 10% and absorbed twice the amount of energy (∼0.68 MJ/m^3^) compared with fibers from straight PNFs (0.30 kJ/m^3^) before fracture (Figure 4b). The high modulus of the straight-PNF-derived microfiber is suggested to originate from the higher modulus of straight PNFs compared to that of curved fibrils indicated by persistence length, as described above. The higher degree of alignment of straight PNFs in the microchannel could also contribute to a higher modulus of the final fiber.^16,19^ It has been shown that straight PNFs form more aligned structures along the flow channel than the curved fibrils at the same focusing condition.^17^ In contrast, the higher strain at break (10%) observed for the curved-PNF fiber indicates a higher tendency of these fibrils to entangle/aggregate than the straight fibrils. The different mechanical behavior resulting from the different morphologies of the fibrils also provides ways to harvest desired properties of the microfiber by varying the composition of these two types of fibrils in the suspension.

**Figure 5:**
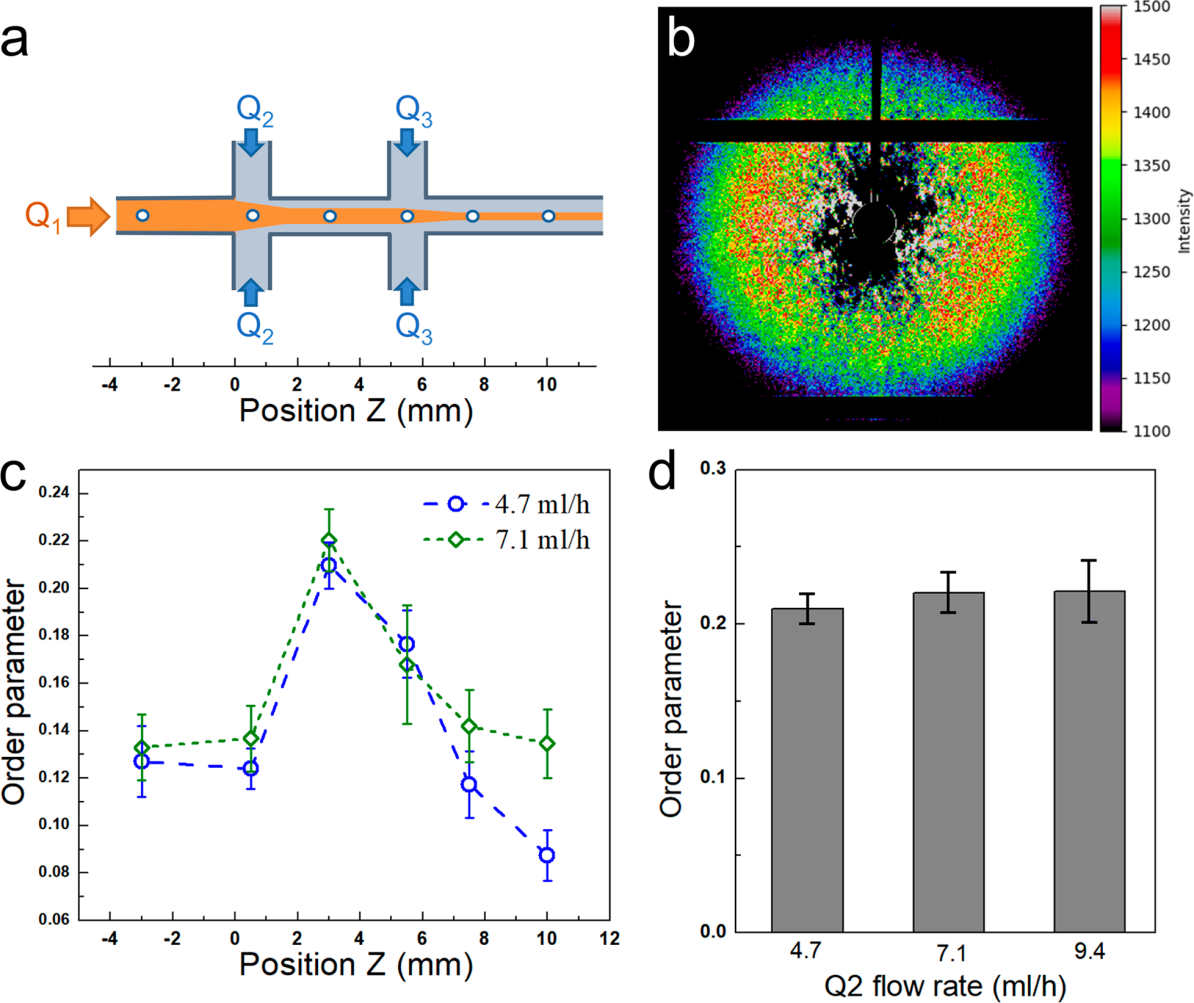
(a) Schematic of the channel geometry employed for the SAXS experiments. White circles show the different downstream positions at the center of the channel where *in situ* SAXS measurements were carried out. (b) Example of SAXS pattern at the location where the strongest fibril alignment is observed. (c) Local order parameters calculated from SAXS patterns with Q2 flow rates of 4.7 and 7.1 mL/h. (d) The greatest order parameter, *i*.*e*., at position *Z* = 3 mm, as a function of Q2 flow rate. The given order parameter is an average value of 10 measurements.

It is noteworthy to mention that the fibers spun from curved PNFs at the higher Q3 sheath rate (33.9 mL/h) have lower modulus but are more extensible than the fibers assembled at a Q3 rate of 24.9 mL/h (Figure 4a). This is in opposition to previous observations of fiber assembly from cellulose nanofibrils or straight PNFs, as increased sheath flow rate results in higher acceleration of the core and typically results in a higher degree of fibril alignment and a higher fiber modulus.^16,21,22,39^ To further address this contradiction, *in situ* small-angle X-ray scattering (SAXS) measurements of the curved fibril alignment in the channel, under different flow conditions, were employed. Due to the low signal/noise ratio downstream of the second sheath flow (Q3), reliable data on the effect of the Q3 flow rate could not be obtained. Instead we analyzed the behavior when altering the Q2 flow rate. The results in Figure 5 show that the order parameter of PNFs in the channel was relatively low (0.2) and did not increase with increased Q2 flow rate. This indicates that the effects in spinnability and modulus that we observe do not originate from fibril alignment but other, presently unknown, aspects of the structure created during hydrodynamic assembly. One explanation could be that the higher ejection rate results in a less dense hydrogel fiber, which reduces the number of genipin cross-links. The dried fiber obtained at the higher Q3 flow rate is indeed slightly thinner (*vide infra*).

**Figure 6:**
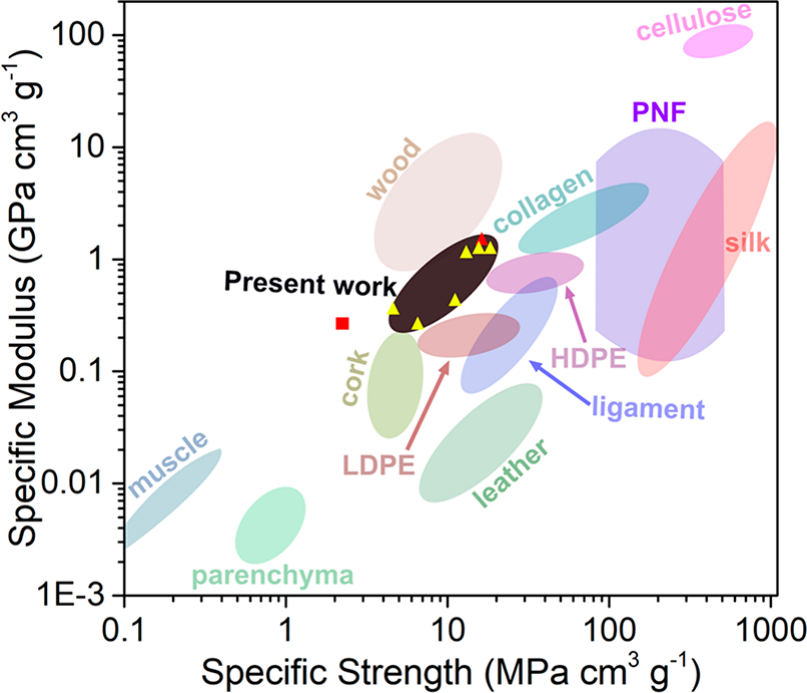
Mechanical properties of bio-based and synthetic materials are displayed as a plot of specific modulus *versus* specific strength. The data of the PNF microfiber fabricated in the present work are illustrated as triangles in the black region in the figure, where the yellow and the red refer to the fibers from curved and straight fibrils, respectively. The red square represents the mechanical properties of the fiber produced by Kamada *etal*.^17^

The strength and modulus of the microfiber, illustrated as yellow (curved PNFs) and red (straight PNFs) triangles in the black area in Figure 6, vary almost 1 order of magnitude due to the change in the morphology of the PNF building blocks, the amount of cross-linker, and the flow-focusing parameters (Figure 6). The wide range in mechanical values in the presence of different amounts of genipin emphasizes the key role of the interactions between fibrils in controlling the mechanical properties of microscale materials. The microfibers fabricated in the present work preserved the modulus of individual PNFs after the microfluidic spinning process. The fibers have specific moduli in the range similar to ligaments,^40^ which is comparable to silk fiber and overall higher than synthetic low-density polyethylene (LDPE); see Figure 6.^41,42^ The specific strength is equivalent to that of wood and higher than most natural elastomers, *e*.*g*., muscle, cork, and leather.^41^

### Morphology of the Dried Microfiber

**Figure 7:**
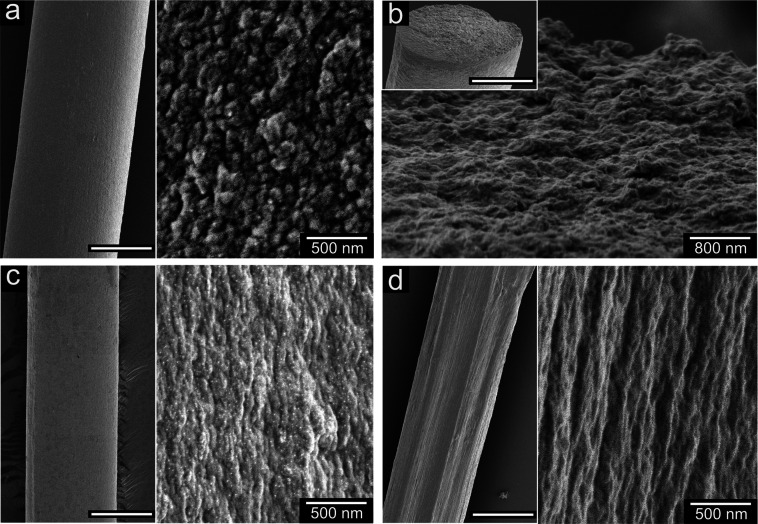
SEM images of the surface (a) and the tensile-fracture cross-section (b) of the microfibers assembled from curved PNFs solely. Surface of the fiber assembled from curved PNFs (c) and straight PNFs (d) with 10 wt % of genipin at a sheath rate of 33 mL/h. The scale bars in the left/inset images are 20 μm.

The air-dried fibers made from curved PNFs with (not shown) and without genipin (Figure 7a) showed a smooth surface with a constant diameter of 35 ± 2 μm along the fiber direction. A closer view of the surface showed that the PNFs assembled into closely packed graupel-like units with a size of 50–200 nm (Figure 7a), similar to the structure observed previously in the assembly of whey PNFs^17^ and recombinant spider silk proteins.^43^ The addition of genipin did not significantly affect the surface morphology of the final fibers, as granular PNF aggregates were also observed on the surface of the cross-linked fibers (Figure S6a). The micrograph of the fiber cross-section after the tensile test demonstrates a rough fracture surface (Figures 7b and S5b). The homogeneous cross-section of the cross-linked fiber (Figure S6b) indicates a good distribution of genipin within the fiber, which avoids different failure behavior along the transversal direction under stress. Nanosized fibril-like objects, presumably originating from stretched fibrils/fibril aggregates, were also observed in the cross-sections of the fibers cross-linked with 5 and 10 wt % genipin (Figure S6c), in accordance with the observed high strain and energy at break of these fibers compared to those with a lower amount of genipin.

The diameter of the dried fibers assembled at the higher sheath flow rate (33 mL/h) was 30 ± 4 μm, slightly smaller than the value of the fiber studied previously (35 ± 2 μm). The surface of the fiber assembled from curved PNFs remained smooth regardless of the second sheath flow rate. A magnified view showed close-packed oval-shaped aggregates with the long axis orientated parallel to the fiber direction (Figure 7c). Compared to the graupel-like units observed previously, the elongated unit (due to the increased sheath rate) may detach more easily from the neighbor units under stress and result in a lower stiffness value (Figure 4). In contrast to the fiber composed of curved PNFs, the fiber from straight PNFs did not have a perfect cylinder shape after drying (Figure 7d). Moreover, the straight PNFs did not aggregate into graupel-like units during the assembling of the microfiber. Instead it showed a texture in agreement with a fiber with aligned constituents, commonly observed in the fibers assembled from cellulose nanofibrils *via* a similar microfluidic method.^19,21,44^ The different morphologies of the microfiber assembled from the two types of fibrils indicated fundamental differences in assembly mechanisms, which resulted in the different mechanical performance of the two fibers described in Figure 4.

## Conclusions

By introducing a bio-based cross-linker, the morphological and mechanical characterization of spun protein microfibers could be expanded beyond previous limitations, including elucidation of the role of the PNF nanostructures on fiber properties. The fibers spun from a PNF suspension containing genipin resulted in a dark green color in less than 2 h (for 10 wt % genipin), indicating extensive cross-linking reactions, confirmed by IR spectroscopy results. An increasing amount of added genipin directly correlated with the mechanical integrity and properties of the fiber. The Young’s modulus reached *ca*. 1.4 GPa at 2 wt % genipin, and the energy at break increased to 0.8 MJ/m^3^ at 10 wt %, more than 4 times the values for the fibers from pure PNFs. With 10 wt % of genipin, the modulus and the stress at break of the microfiber from straight PNFs reached 1.6 GPa and 20 MPa, respectively, which is *ca*. 4 times that of the fiber from curved PNFs spun at the same condition. Thus, it has been demonstrated that the mechanical properties of the PNF fibers can be improved by solely adding a bio-based and nontoxic cross-linker. In addition, the results herein showed that the fibril morphology and the flow rate used for spinning are also critical for defining the mechanical performances of the spun fibers.

## Methods

### Preparation of PNFs

Whey protein isolate (Arla Food Ingredients) was dissolved in 0.1 M HCl (Fisher Scientific) to a concentration of *ca*. 200 g/L. The WPI solution was dialyzed against 0.01 M HCl for 24 h at room temperature using a membrane (Spectrum Laboratories) with a 6–8 kDa molecular weight cutoff to remove salts and small molecules. The HCl solution was changed three times during the dialysis. The dialyzed WPI solution (pH 2) was then diluted to *ca*. 80 or 40 g/L and incubated at 90 °C for 3 days to form curved and straight PNFs, respectively.^6,17^ The obtained PNFs were purified by dialysis against 0.01 M HCl for 3 days using a 100 kDa molecular weight cutoff membrane. Furthermore, the solution of straight fibrils was concentrated by centrifugal membrane filtration with a 100 kDa cutoff. The concentration of the final PNF solution before spinning was determined by measuring the dry weight of the lyophilized materials and was found to be *ca*. 20 g/L. Different concentrations of genipin (Zhixin Biotechnology, China), 1, 2, 5, and 10 wt % with respect to the dry mass of PNFs, were used to prepare the spinning suspensions for the production of a cross-linked microfiber. The genipin was ground before adding it to the PNF samples, and the suspension was then vortexed for 1 min before spinning.

### Spinning of Protein Fibers

The PNF microfibers were formed using the flow setup described in Figure S1.^17^ The flow setup contains three syringe pumps, a flow-focusing channel, and a bath for collecting microfibers. The pumps were used to control the flow rates of the core flow and the first and second sheath flows, which were set to 4.1, 4.7, and 24.9 mL/h, respectively. The rate of the second sheath flow was increased to 33.9 mL/h for the spinning of straight PNFs to avoid clogging the channel. The increased flow rate in the second sheath was also applied to curved fibrils for comparison. The flow channel with a width of 1 mm was milled in a 1-mm-thick stainless-steel plate. The stainless-steel plate was sandwiched between two poly(methyl methacrylate) plates and two aluminum plates. The five plates were screwed together to prevent leakage. In the first sheath flow, pure water was used to detach the core flow from the walls and assist the alignment of fibrils, while acetate buffer (pH 5.2) in the second sheath flow changed the pH of the PNF suspension to its isoelectric point (pH 5.2) to trigger gelation and lock the aligned PNFs. The spinning process was finished within 20 min after adding the genipin to prevent the cross-linking of PNFs in the flow channel. After the spinning, the PNF fibers in the acetate bath were placed in an oven at 50 °C for 14 h to accelerate the cross-linking reaction.

### Atomic Force Microscopy

The morphology of the PNFs was investigated by a multimode 8 atomic force microscope (Bruker Corp., USA) operated in Scanasyst-air mode. The PNF solution was diluted in 0.01 M HCl (1:1000), placed on a freshly cleaved mica surface, and dried in ambient conditions before the measurement. The images were analyzed using Nanoscope 1.5 software (Bruker).

### Rheology

The oscillation test was performed on the cross-linked and non-cross-linked PNF suspensions using a DHR-2 rheometer (TA Instruments, USA), fitted with a 25 mm diameter stainless steel parallel plate at 25 °C. The cross-linked PNF suspension was prepared by conditioning a well-mixed genipin–PNF suspension (curved fibrils, 16 g/L) at 50 °C for 14 h. The sample was taken out from the oven and placed in an ice–water bath for at least 10 min before the test. The sample was then transferred to the rheometer base plate for amplitude sweep in the strain range of 0.01–100% at a constant frequency of 1 Hz. The viscosity of the straight and curved PNF suspensions (20 g/L) was measured by the rheometer equipped with a 60 mm diameter stainless-steel parallel plate. The rotational flow sweep measurement was performed in the strain rate from 0.01 to 100 s^–1^ at 25 °C.

### Scanning Electron Microscope

The morphology of the PNF microfibers was studied by using an S-4800 cold-field-emission scanning electron microscope (Hitachi, Japan) at a voltage of 1 kV. The dried samples were fixed on an aluminum specimen holder using copper double tape and coated with a thin layer of platinum/palladium before the examination in the microscope. Micrographs with a dimension of 2560 × 1920 pixels and a resolution of 512 dpi were recorded.

### Tensile Test

The micromechanical tensile measurements were performed on a Deben Microtest (UK) equipped with a 50 N load cell at a 0.5 mm/min strain rate, using a method based on the work by Andersson *etal*.^45^ The microfibers were dried and conditioned at 50% RH and 23 °C for at least 4 days before the tests. The diameter of the fibers was measured by an optical microscope and was typically around 30 to 35 μm for the fibers spun from PNFs. Before testing, the ends of one single fiber were glued on a piece of paper with an 8–10 mm span length. The whole assembly was then firmly mounted between the grips on the tester stage. The side panel of the paper was cut before the testing (Figure S7). Ten specimens were tested for each sample. The stress–strain curve of each specimen was recorded, and the energy at break of the specimen was calculated from the integral area under the curve before fracture.

### FTIR

The cross-linked and non-cross-linked PNF suspensions were freeze-dried for at least 12 h before the FTIR measurements. The measurements were performed using a PerkinElmer Spotlight 400 FTIR (USA) equipped with a Golden Gate (Specac Ltd., UK) single-reflection ATR crystal. The spectra were recorded between 4000 and 750 cm^–1^ with 16 scans and 4 cm^–1^ resolution.

### *In Situ* Synchrotron SAXS Measurements

Transmission SAXS measurements were performed at the P03 beamline, at PETRAIII storage ring at DESY in Hamburg, Germany.^46^ The measurements were performed with an X-ray wavelength λ = 0.97 Å and sample-to-detector distance of 9035 mm. The scattering patterns were recorded by a single-photon counting detector (Pilatus 1M, Dectris) with the pixel size of 172 × 172 μm^2^. The beam size was 33 × 27 μm^2^ (horizontal × vertical). The same type of flow channel was used for the SAXS study as for the spinning experiments, but the Plexiglas covers were replaced by Kapton films. The flow rates were the same as the ones used for spinning (4.1, 4.7, and 24.9 mL/h for the core flow, the first sheath flow, and the second sheath flow, respectively). The Q2 sheath flow was varied (1.5× and 2× the initial value) to explore the effect on PNF alignment. Alignment order parameters of the PNFs were calculated from the scattering patterns as described in previous work.^17,21,22^

### TGA Measurements

Since the fibers contained too little material for accurate TGA analysis, these measurements were performed using bulk samples. Genipin were added to solutions of curved PNFs to produce samples containing 0%, 5%, and 10% genipin, respectively. The solutions were incubated at 50 °C for 12 h. The resulting solutions were frozen at −35 °C overnight and lyophilized for 72 h. The dry samples were conditioned at 50% RH for 72 h. A piece of the sample was inserted in a TGA instrument directly after removing the material from the RH room (to avoid water losses). The TGA instrument was run from 40 °C at a heating rate of 10 K/min in a nitrogen atmosphere. The settling option was removed so that the TGA would record the weight from the insertion time in the TGA furnace.

## Supporting Information Available

Illustrations of the flow-focusing setup; second derivative FTIR spectra; TGA measurements; AFM analysis of curved and straight PNFs; viscosity of PNF suspensions; additional SEM images of microfibers; illustrations of the sample preparation for the tensile test (PDF)
