# Supplementary material for: Robust Assembly of Cross-Linked Protein Nanofibrils into Hierarchically Structured Microfibers
Source: ACS Nano. 2022 Jul 29;16(8):12471–9. doi: 10.1021/acsnano.2c03790 (PMC9413408; doi:10.1021/acsnano.2c03790)
Supplement: Supplementary file 1 — nn2c03790_si_001.pdf [file nn2c03790_si_001.pdf]

SUPPORTING INFORMATION

Robust Assembly of Crosslinked Protein Nanofibrils into Hierarchically Structured Microfibers

Xinchen Ye[1], Antonio J. Capezza[1], Saeed Davoodi[2], Xin-Feng Wei[1], Richard L. Andersson[1], Andrei Chumakov[3], Stephan V. Roth[1,3], Maud Langton[4], Fredrik Lundell[2*], Mikael S. Hedenqvist[1*], Christofer Lendel[5*]

[1] Department of Fibre and Polymer Technology, KTH Royal Institute of Technology, Teknikringen 56-58, SE-100 44, Stockholm, Sweden.

[2] Department of Engineering Mechanics, KTH Royal Institute of Technology, Teknikringen 8, SE-100 44, Stockholm, Sweden.

[3] Deutsches Elektronen-Synchrotron DESY, Notkestr. 85, D-22607 Hamburg, Germany.

[4] Department of Molecular Sciences, SLU, Swedish University of Agricultural Sciences, BioCentrum, Almas allé 5, SE-756 61, Uppsala, Sweden.

[5] Department of Chemistry, KTH Royal Institute of Technology, Teknikringen 30, SE-100 44, Stockholm, Sweden.

* Correspondence to: F.L.: frlu@kth.se, M.S.H.: mikaelhe@kth.se, C.L.: lendel@kth.se

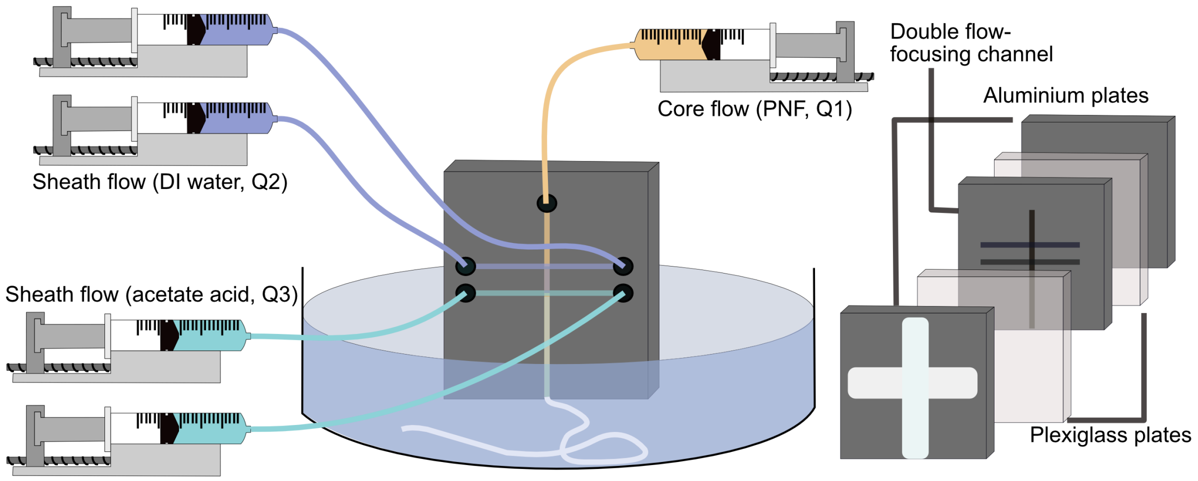

Figure S1. Illustration of the flow-focusing setup.

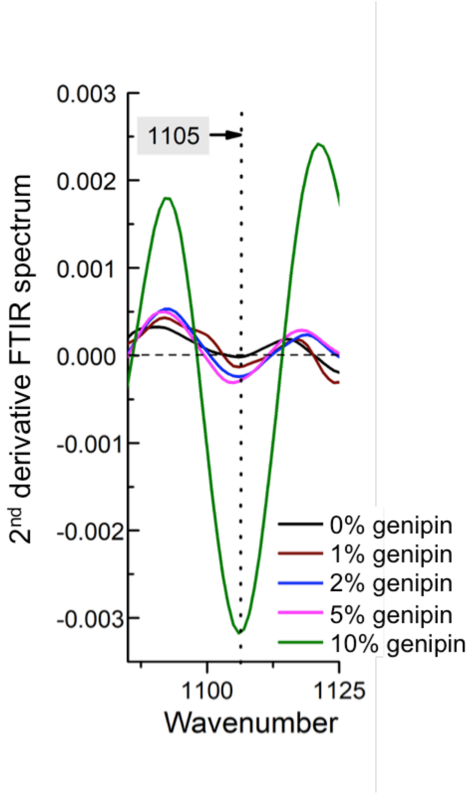

Figure S2. Second derivative of the FTIR spectra displayed in Figure 1b.

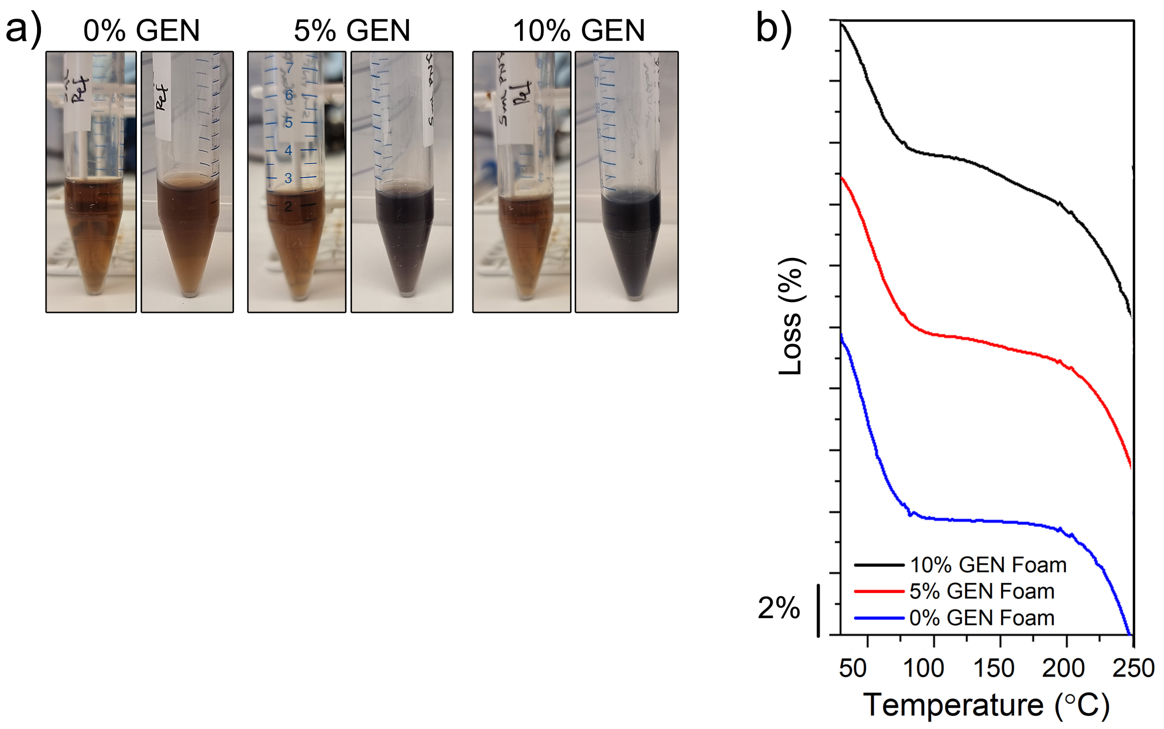

Figure S3. TGA experiments to measure water uptake in PNF samples with and witout genipin. a) Photographs showing the samples before (left) and after (right) reaction with genipin. The samples were lyophilized and conditioned at 50% RH for 72 h before TGA analysis. b) TGA thermograms for PNF samples without (blue), with 5% genipin (red) and 10% genipin (black).

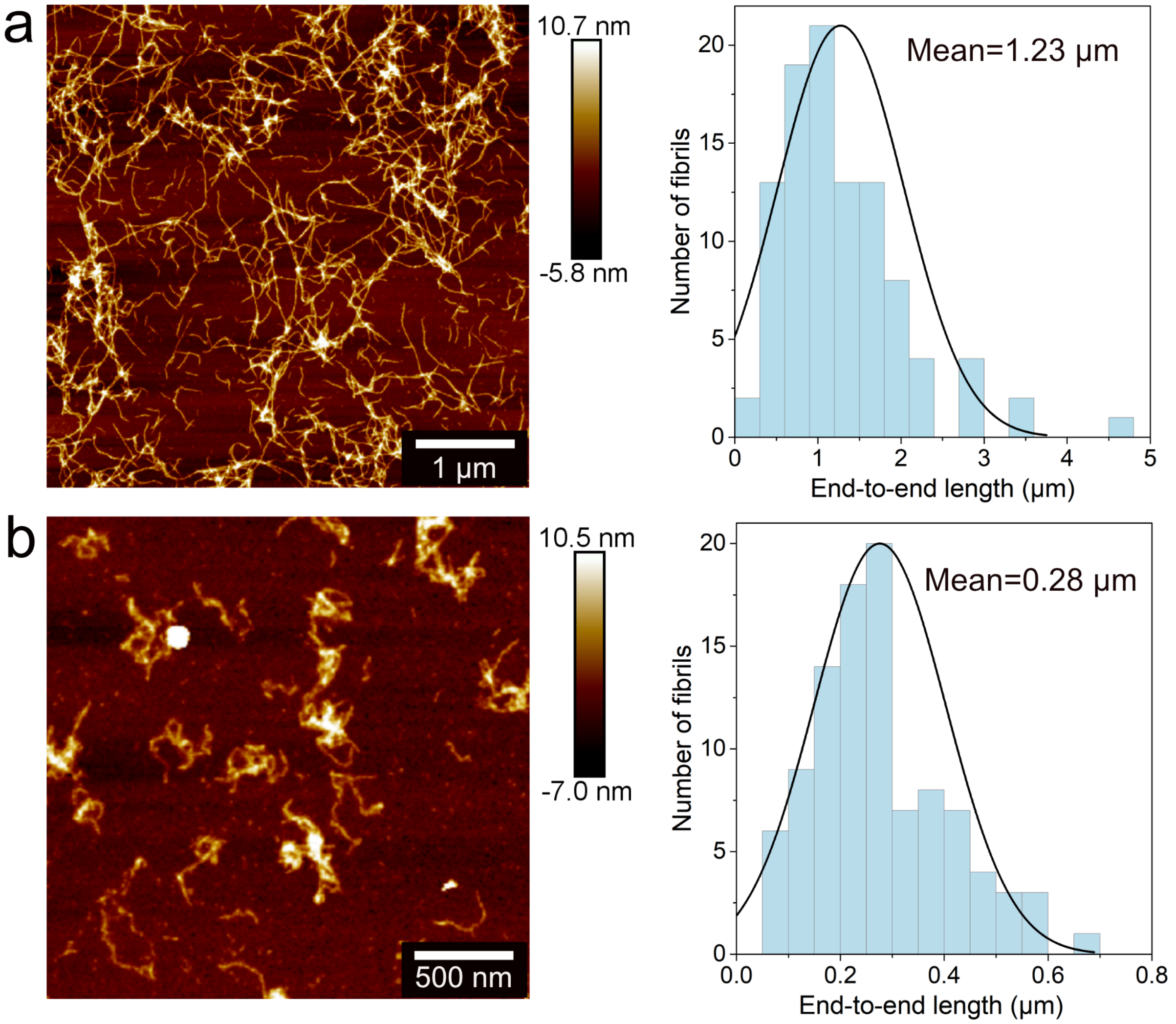

Figure S4. AFM images of the straight (a) and curved (b) fibrils grown from 40 and 80 g/L WPI solutions, respectively.

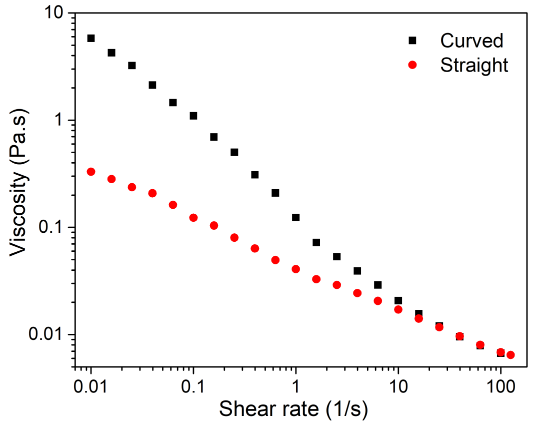

Figure S5. The viscosity of the curved and straight PNF suspension at a PNF concentration of 20 g/L (calculated from dry weight).

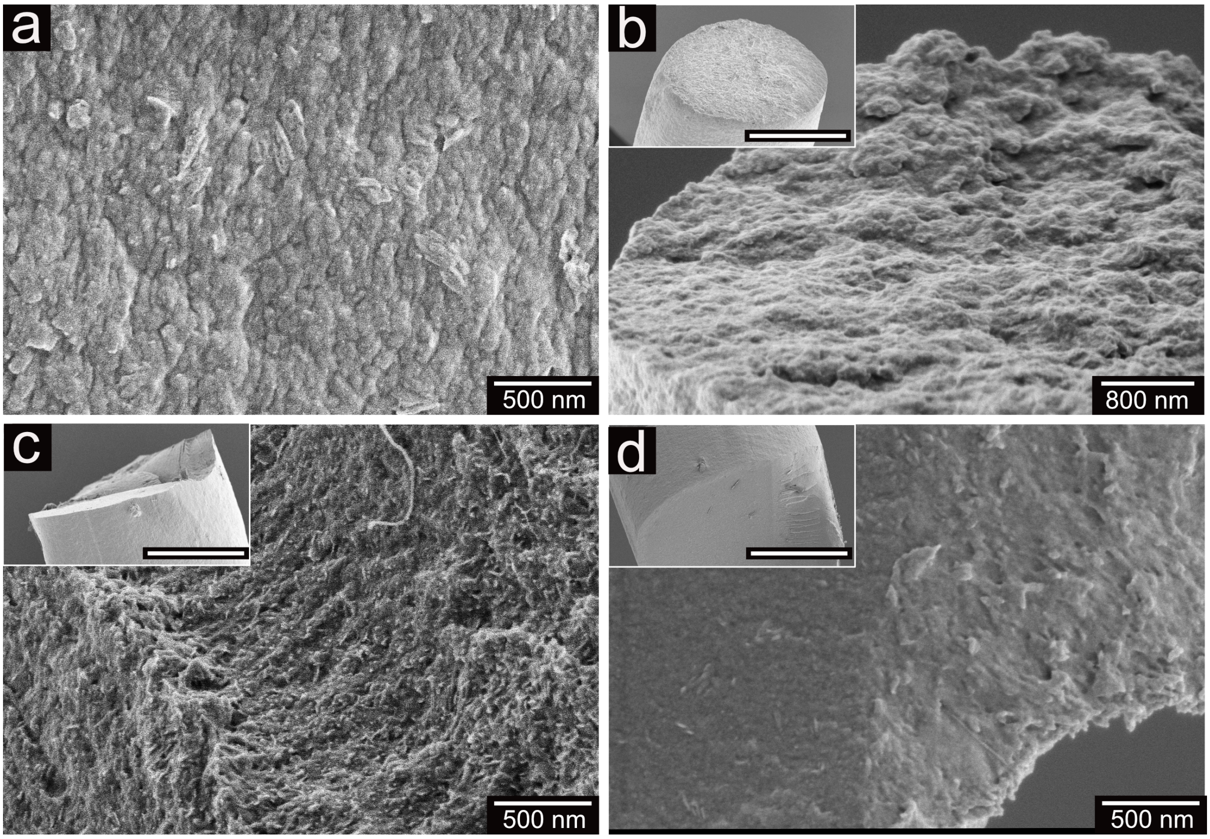

Figure S6. SEM images of the surface (a) and the cross-section (b) of the microfibre with 2 wt% genipin. (c) The cross-section of the fibre crosslinked with 5 wt% genipin. (d) The cross-section of the fibre crosslinked with 10 wt% genipin. The scale bar in the inserted images are 20 µm.

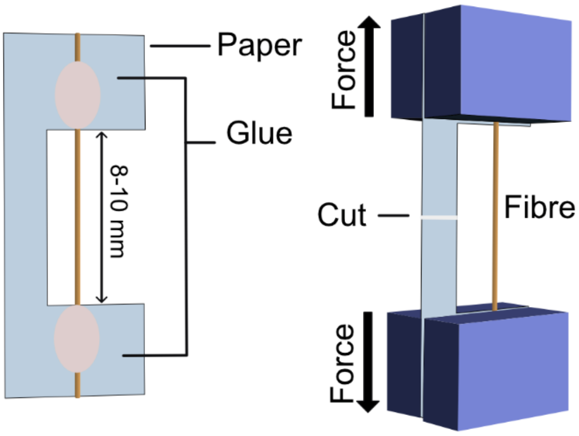

Figure S7. Illustration of the sample preparation for tensile test.
